# *NPPA*-Associated Atrial Dilated Cardiomyopathy: Genotypic and Phenotypic Insights From an Ultrarare Inherited Disorder

**DOI:** 10.1016/j.jaccas.2025.105141

**Published:** 2025-08-21

**Authors:** Cinzia Forleo, Marco Maria Dicorato, Maria Cristina Carella, Paolo Basile, Ilaria Dentamaro, Vincenzo Ezio Santobuono, Andrea Igoren Guaricci, Nicoletta Resta, Marco Matteo Ciccone, Eloisa Arbustini

**Affiliations:** a Cardiology Unit, Interdisciplinary Department of Medicine (DIM), University of Bari Aldo Moro, University Hospital Consortium, Polyclinic of Bari, Bari, Italy; b Internal Medicine Section, Department of Precision and Regenerative Medicine and Ionian Area (DiMePRe-J), University of Bari Aldo Moro, University Hospital Consortium, Polyclinic of Bari, Bari, Italy; c Medical Genetics Unit, Department of Precision and Regenerative Medicine and Ionian Area (DiMePRe-J), University of Bari Aldo Moro, University Hospital Consortium, Polyclinic of Bari, Bari, Italy; d Department of Research, Centre for Inherited Cardiovascular Diseases, IRCCS Foundation, University Hospital Policlinico San Matteo, Pavia, Italy

**Keywords:** atrial dilated cardiomyopathy, atrial paralysis, atrial standstill, autosomal recessive cardiomyopathy, genetic atrial cardiomyopathy, *NPPA* mutation, ultrarare cardiac disease

## Abstract

**Background:**

Atrial standstill is a rare arrhythmogenic disorder characterized by complete atrial electrical and mechanical inactivity. We report the 15th documented case of atrial dilated cardiomyopathy associated with the homozygous c.449G>A (p.Arg150Gln) *NPPA* mutation.

**Case Summary:**

A 31-year-old woman presented with persistent atrial fibrillation, biatrial enlargement, and junctional rhythm. Electrophysiological studies confirmed atrial inexcitability. Despite preserved ventricular function, she required permanent His-bundle pacing. Genetic testing later revealed a homozygous *NPPA* mutation, whereas heterozygous family members remained asymptomatic.

**Discussion:**

This case highlights the diagnostic value of genetic testing in young patients with atrial fibrillation and no structural heart disease. Early recognition of *NPPA*-related atrial dilated cardiomyopathy may guide arrhythmia management and anticoagulation strategies, reducing thromboembolic risk.

**Take-Home Message:**

Broader implementation of genetic screening in selected individuals with isolated atrial dysfunction may support earlier diagnosis, personalized treatment, and better outcomes in this ultrarare condition.

## Graphical Abstract

**Figure:**
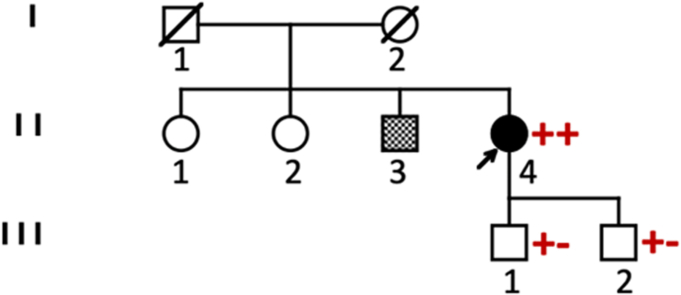

Atrial standstill, also known as atrial paralysis, is a rare myocardial disorder marked by absence of electrical and mechanical atrial activity.^1^ It can be idiopathic or secondary to heterogeneous conditions like neuromuscular dystrophies, laminopathies, cardiac amyloidosis, and Ebstein's anomaly.^1^ Atrial standstill results in unexcitable atrial myocardium replaced by a bradycardic junctional escape rhythm.^2^^,^^3^

**Take-Home Messages**

• In patients with atrial fibrillation, atrial standstill, and biatrial enlargement without structural heart disease, rare genetic etiologies like *NPPA* mutations should be considered.
• This case highlights the value of targeted genetic testing in patients with isolated atrial dysfunction to enable timely diagnosis and personalized management.

The incidence is unknown due to rarity.^2^ The idiopathic familial form of atrial dilated cardiomyopathy (ADCM) has been linked to combined heterozygous sodium voltage-gated channel alpha subunit 5 and Connexin40 mutations, recessive sodium voltage-gated channel alpha subunit 5 mutations, or homozygous *NPPA* mutations.^1^ Disertori et al^1^ first identified the homozygous c.449G>A (p.Arg150Gln) *NPPA* mutation in 13 patients. Recently, Silva et al^4^ identified the same pathogenic variant in a patient with persistent atrial fibrillation (AF) and fibrotic atrial cardiomyopathy. To our knowledge, we describe the 15th documented case worldwide of ADCM associated with a homozygous *NPPA* mutation. Our findings underscore the importance of integrating atrial dysfunction into heart failure (HF) screening strategies to enable earlier detection and tailored management of this ultrarare condition.

## History of Presentation

**Figure 1:**
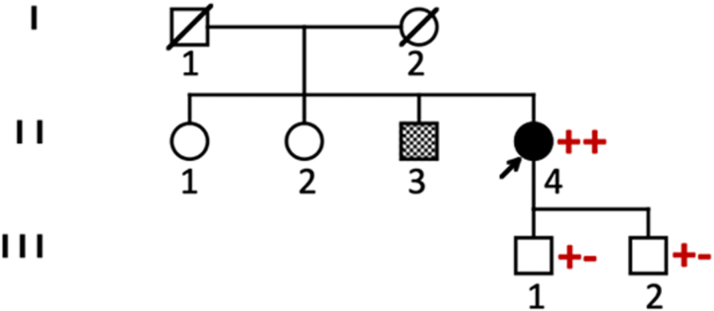
Pedigree of the Proband Affected by ADCM Due to a Homozygous *NPPA* c.449G>A (p.Arg150Gln) Mutation (++) Both parents (I:1 and I:2) are deceased; clinical history suggests maternal chronic heart failure. The proband's 2 sisters (II:1 and II:2) are clinically unaffected and chose not to have genetic testing. Her brother (II:3) carries a pacemaker, but genetic status is unknown. The proband's 2 sons (III:1 and III:2) are heterozygous carriers of the *NPPA* variant (+−) and are asymptomatic with no signs of ADCM. Symbols are as follows: arrow = proband, squares = males, circles = females, diagonal line = deceased, solid fill = affected, shaded square = unclear cardiac phenotype, red symbols = *NPPA* genotype. ADCM = atrial dilated cardiomyopathy.

**Figure 2:**
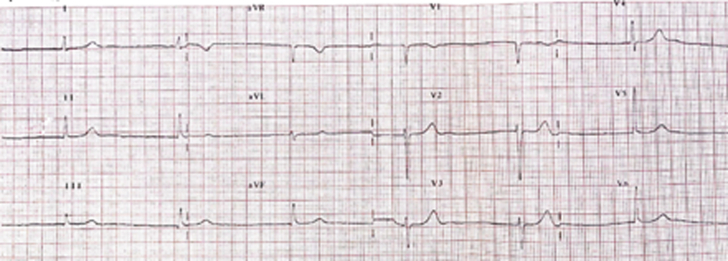
Baseline 12-Lead Electrocardiogram Showing Absence of P Waves and Atrial Electrical Activity, Consistent With Atrial Standstill The rhythm is a regular junctional escape rhythm at 40 beats/min.

A 31-year-old woman, with an unspecified family history of maternal chronic HF (Figure 1, Table 1), presented with abdominal pain. Her baseline electrocardiogram (ECG) showed AF with low-voltage F waves and a junctional escape rhythm at 40 beats/min (Figure 2); echocardiography revealed biatrial dilatation and preserved biventricular function. Holter ECG documented persistent AF with junctional rhythm.

**Table 1:** Major Clinical Information on Family Members of the Proband

Family Member	Age, y	Clinics
I:1	67	Death, noncardiac cause
I:2	65	Death, HF, unspecified cause
II:1	59	Healthy, asymptomatic
II:2	56	Healthy, asymptomatic
II:3	46	Few clinical info; PM
II:4, proband	31-48	Atrial standstill (see text)
III:1, III:2	28, 26	Healthy, heterozygous carriers

HF = heart failure; PM = pacemaker.

## Investigation

She was later referred to our cardiology unit. Echocardiography confirmed significant atrial enlargement, with mild valvular regurgitation and preserved biventricular function. Stress testing was negative; cardiac magnetic resonance excluded structural cardiomyopathy. Electrophysiological study (EPS) revealed atrial septal rhythm, right atrium inexcitability, and a supra-Hisian atropine-sensitive first-degree atrioventricular block. Anticoagulation was started.

## Management

**Figure 3:**
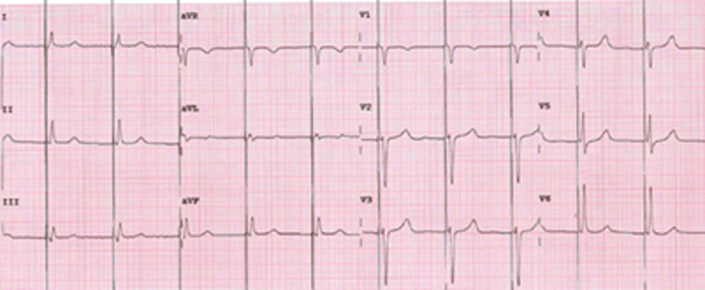
Follow-Up 12-Lead Electrocardiogram of the Proband Demonstrating a Ventricular Rhythm Driven by a His-Bundle Pacemaker Operating in VVIR Mode Atrial electrical activity remains absent, consistent with permanent atrial standstill.

**Figure 4:**
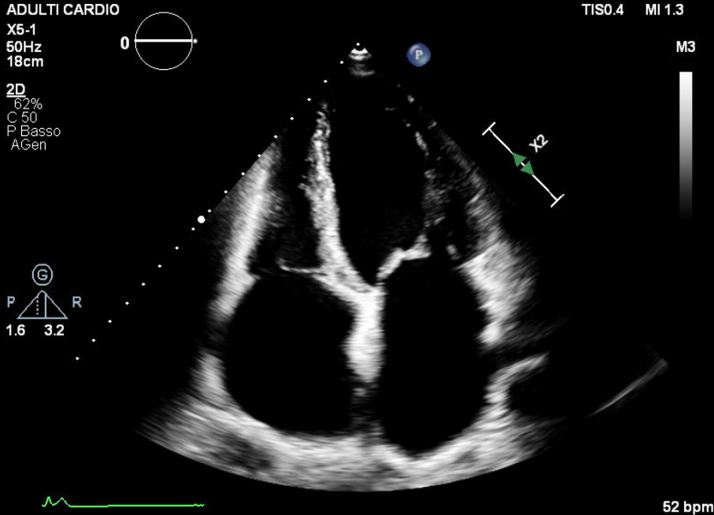
2-Dimensional Transthoracic Echocardiography, Apical 4-Chamber View of the Proband, Showing Marked Biatrial Enlargement With Preserved Biventricular Size and Systolic Function

**Figure 5:**
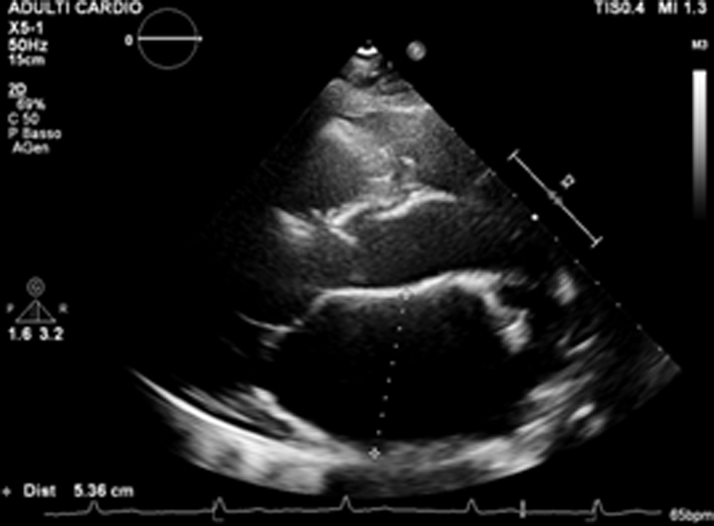
Parasternal Long-Axis Transthoracic Echocardiographic View of the Proband Showing a Markedly Enlarged Left Atrium, With an Anteroposterior Diameter Measuring 54 mm

Two years later, the patient developed worsening symptoms—dyspnea, dizziness, and chest pain. EPS confirmed atrial paralysis with right atrium inexcitability and junctional rhythm. No areas of low voltage were documented. A permanent pacemaker with His bundle pacing was implanted (Figure 3). Postoperative Holter ECG showed continuous ventricular pacing and absent atrial activity. The patient was followed every 6 months, and periodic echocardiography showed stable left ventricular function. Troponin and N-terminal pro–B-type natriuretic peptide remained normal. Approximately 10 years later, she reported palpitations and dyspnea for moderate efforts; device interrogation detected nonsustained ventricular tachycardia. Although the patient had preserved left ventricular systolic function, a multidrug regimen was initiated due to symptoms of exertional dyspnea, elevated right-sided pressures, biatrial enlargement with impaired atrial reservoir function, and evidence of volume overload. These findings are consistent with heart failure with preserved ejection fraction pathophysiology in the context of atrial standstill. Treatment was therefore guided by international HF guidelines, leading to clinical improvement.^5^ The most recent echocardiogram showed severe biatrial dilatation (left atrium diameter: 54 mm; left atrium volume index: 73 mL/m^2^; right atrium area: 50 cm^2^), dilated inferior vena cava (22 mm) with reduced collapse, and elevated right systolic pressure (60 mm Hg). Ventricular size and function were normal (left ventricular ejection fraction: 55%) (Figures 4 and 5, Video 1).

## Outcome and Follow-Up

At 48 years of age, after obtaining the patient's written informed consent, blood samples were taken for molecular analysis of 128 genes associated with arrhythmias and cardiomyopathies. The pathogenic homozygous Arg150Gln (c.449G>A) variant in exon 2 of the *NPPA* gene (NM_006172.3) was identified. Genetic analysis of deceased parents could not be performed: the mother died at 65 years of age due to unspecified congestive HF, and the father died at 67 years of age for noncardiac death (malignancy). We have limited clinical information on her 46-year-old brother who carries a pacemaker, and on the 2 asymptomatic, clinically unaffected sisters (59 and 58 years of age) who chose not to have genetic testing. Both proband's sons (28 and 26 years of age) are heterozygous carriers of the maternal *NPPA* variant, in sinus rhythm and asymptomatic without ADCM-related traits, demonstrating that only the homozygous mutation causes the phenotype.

## Discussion

AF in young patients without structural heart disease is rare and warrants thorough investigation to identify underlying causes. A genetic predisposition in atrial standstill is suggested by pathogenic *NPPA* variants, first reported by Disertori et al.^1^^,^^3^ Already in 1983, they described an 8-year follow-up of atrial dilatation progressing to standstill in 8 patients, later expanded to 13 individuals from the same area in northeastern Italy.^1^ Our patient has been a native of a geographic area in Southeastern Italy for at least 4 generations. She is not aware of distant relatives living in the Northeastern Italy area where the previously described Italian patients originate.

Disertori et al^1^^,^^3^ outlined the natural history of idiopathic atrial dilatation evolving to standstill and identified a homozygous *NPPA* missense variant (p.Arg150Gln) in all affected individuals from 6 families.^1^^,^^3^ The disease was characterized by adult-onset, severe biatrial enlargement, early supraventricular arrhythmias with progressive atrial electrical inactivity, thromboembolic events, and preserved left ventricular function. Homozygous individuals showed markedly reduced midregional proatrial natriuretic peptide levels, whereas heterozygous carriers remained asymptomatic with normal atrial natriuretic peptide (ANP) levels. This defines autosomal recessive ADCM as a rare fibrotic atrial cardiomyopathy *NPPA*-linked mutation and ANP deficiency.^1^^,^^3^

More recently, Silva et al^4^ evaluated a young patient with the same homozygous pathogenic variant in the *NPPA* gene. This patient presented with asymptomatic persistent AF, preserved left ventricular function, extensive atrial fibrosis on cardiac magnetic resonance, and extensive areas of low voltage on EPS.

Diagnosing ADCM can be challenging in patients with AF and progressive atrial dysfunction. The diagnosis of permanent atrial standstill is crucial for prognosis and should be suspected in patients with absent P waves and regular R-R intervals on ECG.^1^^,^^2^^,^^6^ The presence of a junctional escape rhythm supports the diagnosis. Echocardiography typically shows biatrial enlargement. ANP levels can be helpful but are not diagnostic. When noninvasive findings suggest atrial standstill, intracavitary EPS remains the gold standard for confirmation.^1^^,^^2^ Clinically, atrial standstill can be asymptomatic or presents with variable nonspecific symptoms such as palpitations, syncope, or dyspnea. Atriomegaly-related thromboembolic events are common and can be the first manifestation, sometimes resulting in sudden cardiac death.^2^^,^^6^ The identification of a pathogenic homozygous Arg150Gln variant in the *NPPA* gene sheds light on the genetic basis of atrial disease, influencing arrhythmia management and disease progression.^7^ Our case highlights the need for broader consideration of genetic testing in patients with unexplained atrial dysfunction and arrhythmias with implications for treatment strategies.

## Conclusions

ADCM due to the homozygous *NPPA* p.Arg150Gln mutation is an ultrarare condition with severe atrial dysfunction and high thromboembolic risk. We describe the 15th reported patient with this mutation. Our study confirms that ADCM occurs only in homozygotes, whereas heterozygous carriers are asymptomatic. Although we could not prove that our patient comes from the same region described by Disertori et al,^1^^,^^3^ she shares the same mutation and clinical features; her sons are heterozygous carriers without ADCM signs.

Further studies are needed to elucidate *NPPA*-related mechanisms and to evaluate targeted therapies, including modulation of natriuretic peptide signaling. Early genetic screening in selected populations may enable timely diagnosis, prevent HF, and guide appropriate management.

## Funding Support and Author Disclosures

The authors have reported that they have no relationships relevant to the contents of this paper to disclose.

## Appendix

Video 1 Three-Dimensional Echocardiographic Assessment Showing Preserved Left Ventricular Ejection Fraction and Volumes The same modality confirms left atrial enlargement and dysfunction.
